# Supplementary material for: OTX2 controls chromatin accessibility to direct somatic versus germline differentiation
Source: EMBO Rep. 2025 Dec 8;27(2):341–66. doi: 10.1038/s44319-025-00622-2 (PMC12852747; doi:10.1038/s44319-025-00622-2)
Supplement: Supplementary file 3 — Expanded View Figures [file 44319_2025_622_MOESM3_ESM.pdf]

Expanded View Figures

Figure EV1. Characterization of OTX2-bound regions.

(**A**) Tracks of two replicates of OTX2 CUT&RUN at the *Tet2* and *Fgf5* loci. (**B**) Genomic distribution of OTX2-bound regions; promoters are defined as $+/-$ 1 kb from a TSS. (**C**) Heatmap of H3K4me1 (pink) and H3K27ac (blue) signal at ESC-specific, common and EpiLC-specific OTX2-bound distal regions. (**D**) Motif analysis in ESC-specific, common and EpiLC-specific regions. (**E**) Gene ontology of the closest genes to ESC-specific and EpiLC-specific OTX2-bound regions.

© The Author(s)

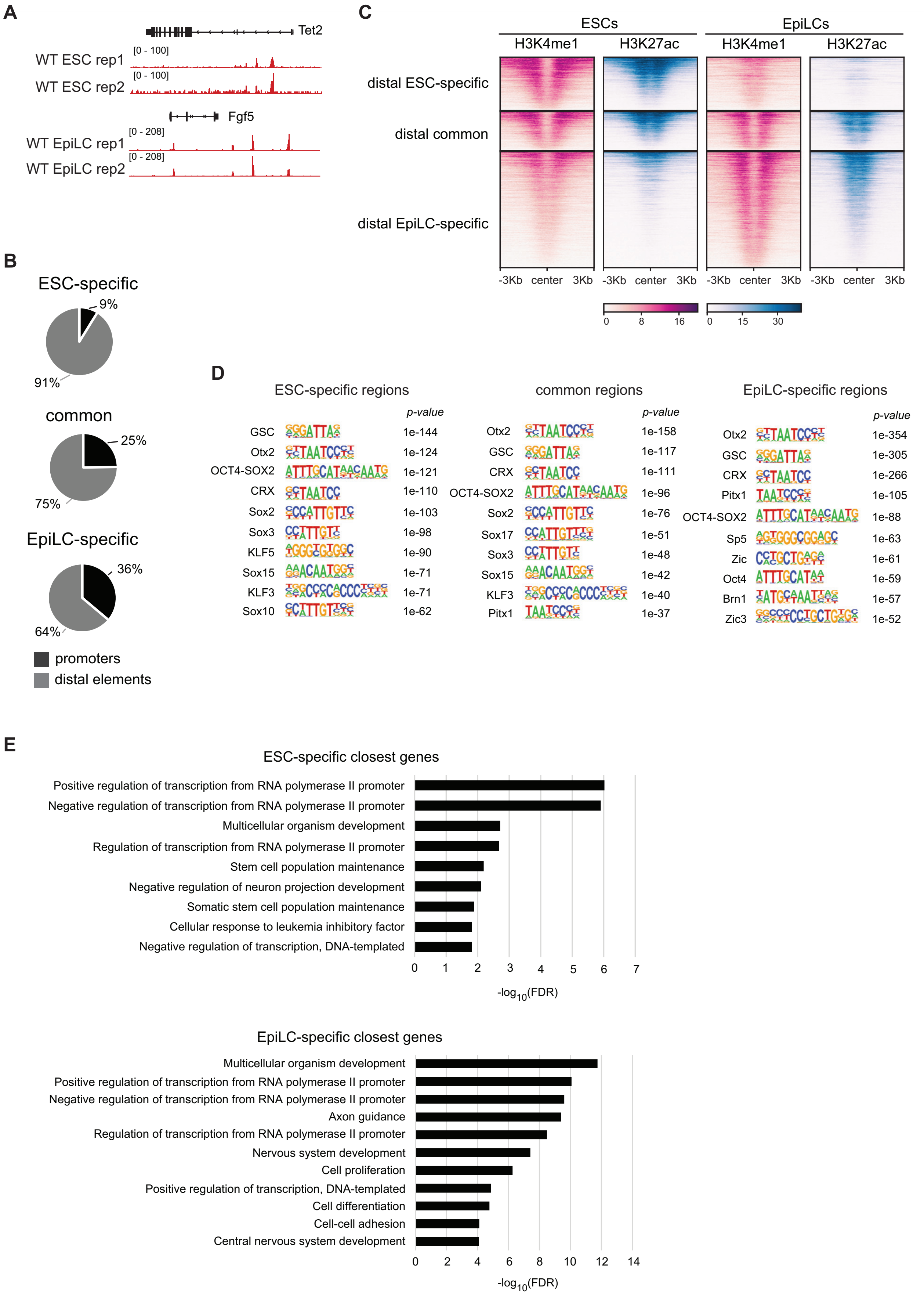
A
Tet2
WT ESC rep1
[0 - 100]
WT ESC rep2
[0 - 100]
Fgf5
WT EpiLC rep1
[0 - 208]
WT EpiLC rep2
[0 - 208]
B
ESC-specific
9%
91%
common
25%
75%
EpiLC-specific
36%
64%
promoters
distal elements
C
ESCs
EpiLCs
H3K4me1
H3K27ac
H3K4me1
H3K27ac
distal ESC-specific
distal common
distal EpiLC-specific
-3Kb center 3Kb
0 8 16
0 15 30
D
ESC-specific regions
p-value
GSC 1e-144
Otx2 1e-124
OCT4-SOX2 1e-121
CRX 1e-110
Sox2 1e-103
Sox3 1e-98
KLF5 1e-90
Sox15 1e-71
KLF3 1e-71
Sox10 1e-62
common regions
p-value
Otx2 1e-158
GSC 1e-117
CRX 1e-111
OCT4-SOX2 1e-96
Sox2 1e-76
Sox17 1e-51
Sox3 1e-48
Sox15 1e-42
KLF3 1e-40
Pitx1 1e-37
EpiLC-specific regions
p-value
Otx2 1e-354
GSC 1e-305
CRX 1e-266
Pitx1 1e-105
OCT4-SOX2 1e-88
Sp5 1e-63
Zic 1e-61
Oct4 1e-59
Brn1 1e-57
Zic3 1e-52
E
ESC-specific closest genes
Positive regulation of transcription from RNA polymerase II promoter
Negative regulation of transcription from RNA polymerase II promoter
Multicellular organism development
Regulation of transcription from RNA polymerase II promoter
Stem cell population maintenance
Negative regulation of neuron projection development
Somatic stem cell population maintenance
Cellular response to leukemia inhibitory factor
Negative regulation of transcription, DNA-templated
0 1 2 3 4 5 6 7
-log10(FDR)
EpiLC-specific closest genes
Multicellular organism development
Positive regulation of transcription from RNA polymerase II promoter
Negative regulation of transcription from RNA polymerase II promoter
Axon guidance
Regulation of transcription from RNA polymerase II promoter
Nervous system development
Cell proliferation
Positive regulation of transcription, DNA-templated
Cell differentiation
Cell-cell adhesion
Central nervous system development
0 2 4 6 8 10 12 14
-log10(FDR)

© The Author(s)

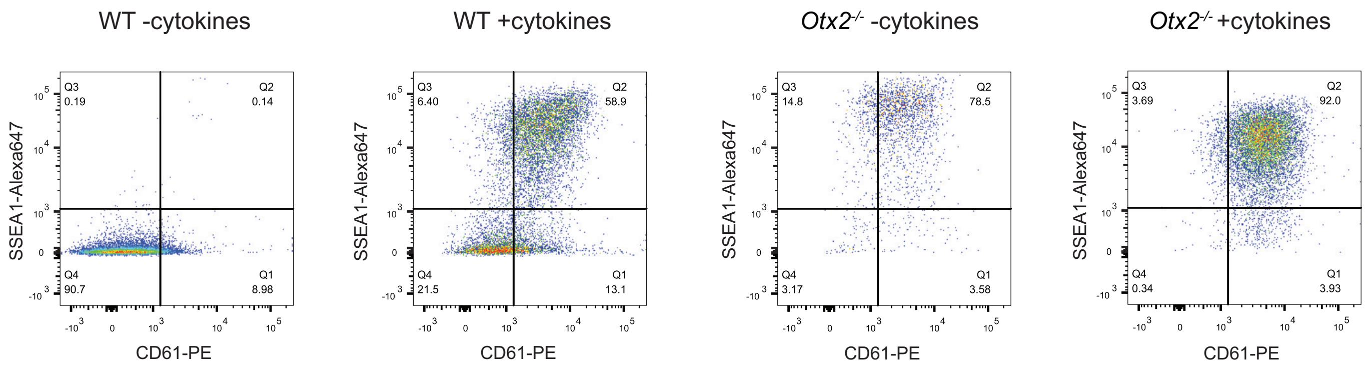

Figure EV2. CD61 and SSEA1 expression at day 6 of PGCLC differentiation.

Flow cytometry plots showing surface expression of CD61 and SSEA1 in d6 aggregates from wild-type and *Otx2*$^{-/-}$ cells cultured in the presence or absence of PGCLC-inducing cytokines.

© The Author(s)

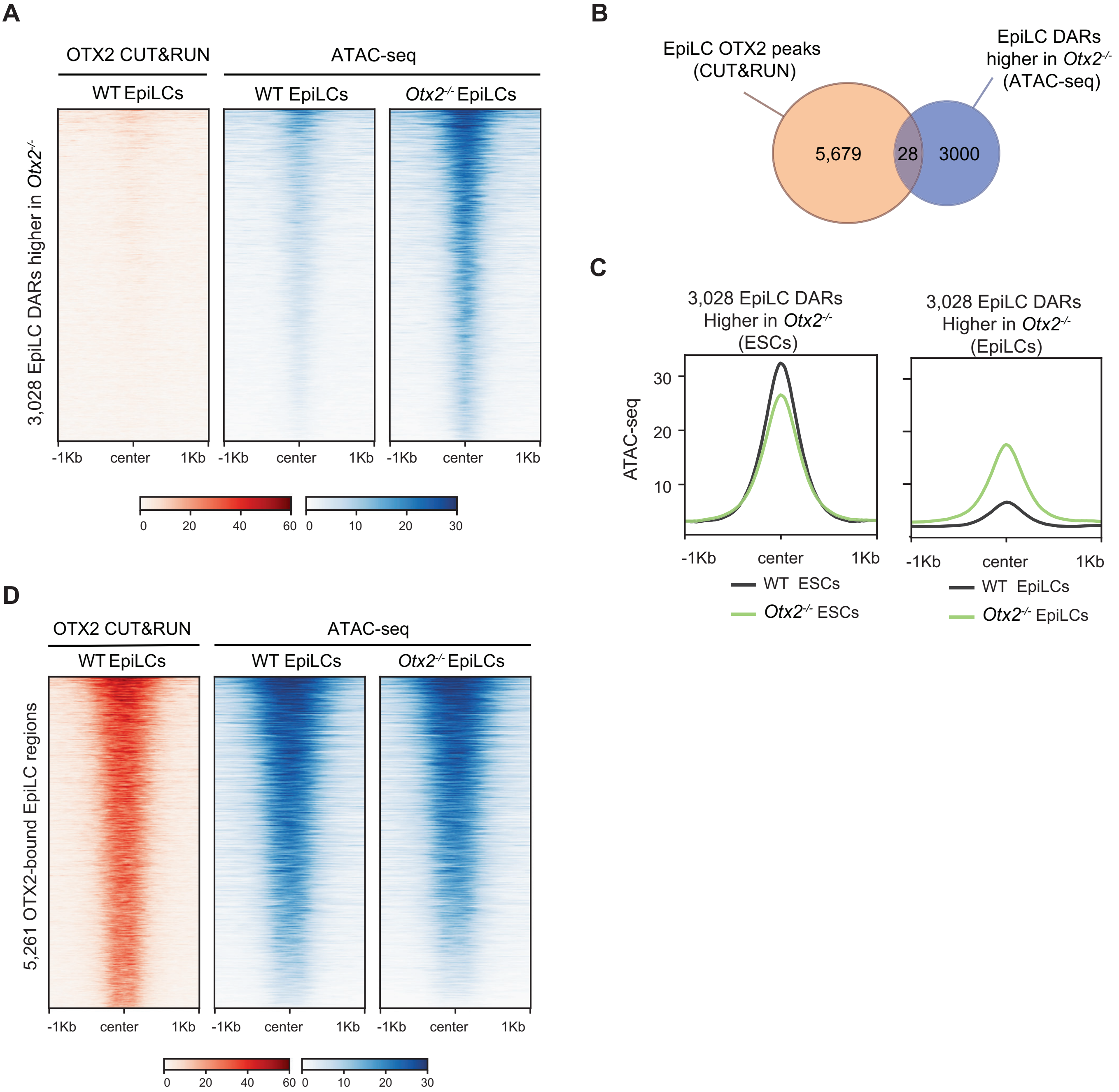

Figure EV3. OTX2 indirectly controls closure of chromatin in the ESC to EpiLC transition.

(**A**) Heatmap of OTX2 binding (CUT&RUN - red) and accessibility (ATAC-seq - blue) at 3028 EpiLC regions that show increased accessibility in *Otx2*$^{-/-}$ EpiLCs. (**B**) Venn diagram of the overlap of OTX2-bound regions in EpiLCs (orange) and EpiLC DARs that are more accessible in *Otx2*$^{-/-}$ EpiLCs (blue). (**C**) Read density profiles of ATAC-seq in wild-type and *Otx2*$^{-/-}$ ESCs (left) and wild-type and *Otx2*$^{-/-}$ EpiLCs (right) at the 3028 EpiLC DARs that are more accessible in *Otx2*$^{-/-}$ EpiLCs. (**D**) Heatmap of OTX2 binding (CUT&RUN - red) and accessibility (ATAC-seq - blue) at 5261 OTX2-bound EpiLC regions that do not show accessibility changes in *Otx2*$^{-/-}$ EpiLCs.

 © The Author(s)

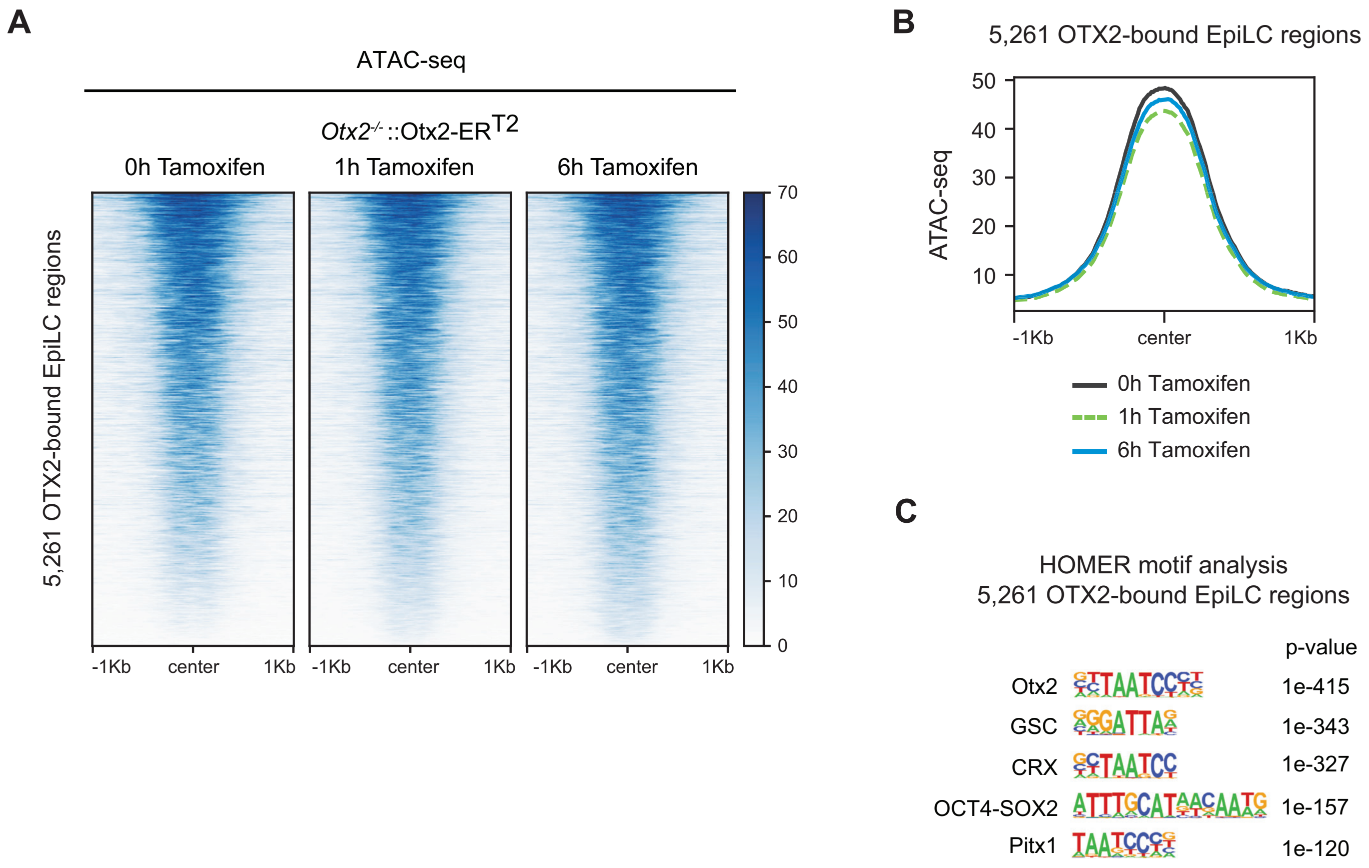

Figure EV4. Most OTX2-bound EpiLC regions do not change accessibility in *Otx2*$^{-/-}$ cells.

(**A**) Heatmap of ATAC-seq signal at the 5261 OTX2-bound EpiLC regions that do not change accessibility in *Otx2*$^{-/-}$::Otx2-ER^{T2} EpiLCs when treated for 1 or 6 h with tamoxifen. (**B**) Average read density profile of ATAC-seq signal at the 5261 OTX2-bound EpiLC regions in *Otx2*$^{-/-}$::Otx2-ER^{T2} EpiLCs treated for 1 h (dashed green) or 6 h (blue) with tamoxifen compared to untreated cells (black). (**C**) Motif analysis in the 5261 OTX2-bound EpiLC regions.

© The Author(s)

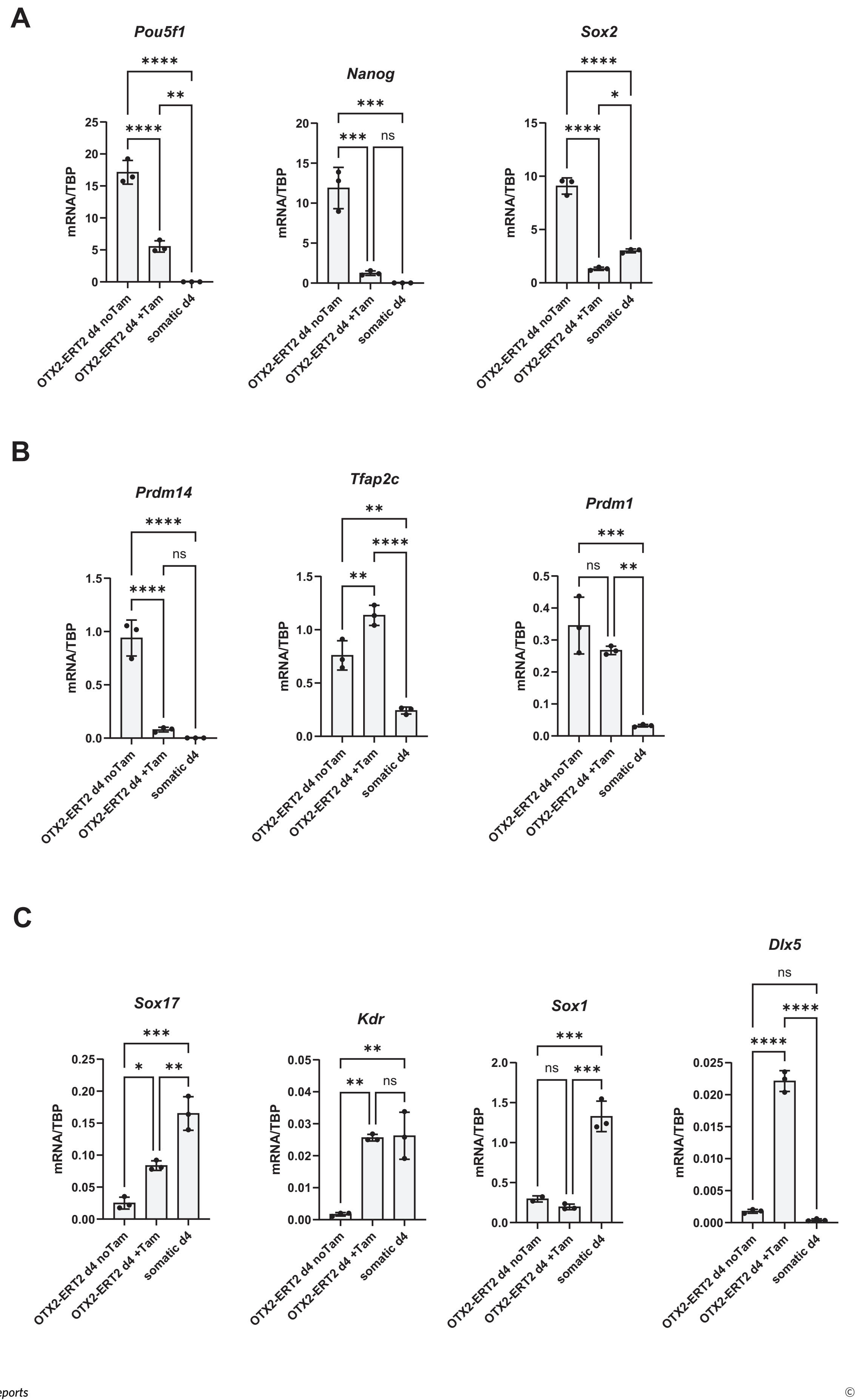
A
Pou5f1
Nanog
Sox2
mRNA/TBP
OTX2-ERT2 d4 noTam
OTX2-ERT2 d4 +Tam
somatic d4
B
Prdm14
Tfap2c
Prdm1
C
Sox17
Kdr
Sox1
Dlx5
ns

 © The Author(s)

Figure EV5. Expression of marker transcripts during differentiation.

mRNA levels of pluripotency-associated markers (**A**), PGC-specific TF (**B**) and somatic-associated markers (**C**) in tamoxifen-treated and untreated *Otx2*$^{-/-}$::Otx2-ER^{T2} cells differentiated for 2 and 4 days in GK15 in the presence of PGC-inducing cytokines or in somatic cells differentiated in GK15 without PGC-inducing cytokines. mRNA levels were quantified by RT-qPCR and normalised to TBP mRNA levels. Data are from a representative of 2 independent experiments (centre: mean, data points: technical triplicates, error bars: standard deviation). The strength of statistical significance was defined using the following thresholding system: ns (not significant) = $P \geq 0.05$; * = $0.01 < P < 0.05$; ** = $0.001 < P < 0.01$; *** = $0.0001 < P < 0.001$; ****$P < 0.0001$. Statistical comparison for *Pou5f1*: OTX2-ERT2 d4 no Tam vs OTX2-ERT2 d4 +Tam $P < 0.0001$; OTX2-ERT2 d4 no Tam vs somatic d4 $P < 0.0001$; OTX2-ERT2 d4 +Tam vs somatic d4 $P = 0.0031$. Statistical comparison for *Nanog*: OTX2-ERT2 d4 no Tam vs OTX2-ERT2 d4 +Tam $P = 0.0003$; OTX2-ERT2 d4 no Tam vs somatic d4 $P = 0.0002$; OTX2-ERT2 d4 +Tam vs somatic d4 $P = 0.6053$. Statistical comparison for *Sox2*: OTX2-ERT2 d4 no Tam vs OTX2-ERT2 d4 +Tam $P < 0.0001$; OTX2-ERT2 d4 no Tam vs somatic d4 $P < 0.0001$; OTX2-ERT2 d4 +Tam vs somatic d4 $P = 0.0102$. Statistical comparison for *Prdm14*: OTX2-ERT2 d4 no Tam vs OTX2-ERT2 d4 +Tam $P < 0.0001$; OTX2-ERT2 d4 no Tam vs somatic d4 $P < 0.0001$; OTX2-ERT2 d4 +Tam vs somatic d4 $P = 0.6214$. Statistical comparison for *Tfap2c*: OTX2-ERT2 d4 no Tam vs OTX2-ERT2 d4 +Tam $P = 0.0080$; OTX2-ERT2 d4 no Tam vs somatic d4 $P = 0.0016$; OTX2-ERT2 d4 +Tam vs somatic d4 $P < 0.0001$. Statistical comparison for *Blimp1*: OTX2-ERT2 d4 no Tam vs OTX2-ERT2 d4 +Tam $P = 0.2377$; OTX2-ERT2 d4 no Tam vs somatic d4 $P = 0.0008$; OTX2-ERT2 d4 +Tam vs somatic d4 $P = 0.0035$. Statistical comparison for *Sox17*: OTX2-ERT2 d4 no Tam vs OTX2-ERT2 d4 +Tam $P = 0.0118$; OTX2-ERT2 d4 no Tam vs somatic d4 $P = 0.0001$; OTX2-ERT2 d4 +Tam vs somatic d4 $P = 0.0023$. Statistical comparison for *Kdr*: OTX2-ERT2 d4 no Tam vs OTX2-ERT2 d4 +Tam $P = 0.0012$; OTX2-ERT2 d4 no Tam vs somatic d4 $P = 0.0010$; OTX2-ERT2 d4 +Tam vs somatic d4 $P = 0.9831$. Statistical comparison for *Sox1*: OTX2-ERT2 d4 no Tam vs OTX2-ERT2 d4 +Tam $P = 0.6755$; OTX2-ERT2 d4 no Tam vs somatic d4 $P = 0.0006$; OTX2-ERT2 d4 +Tam vs somatic d4 $P = 0.0002$. Statistical comparison for *Dlx5*: OTX2-ERT2 d4 no Tam vs OTX2-ERT2 d4 +Tam $P < 0.0001$; OTX2-ERT2 d4 no Tam vs somatic d4 $P = 0.2643$; OTX2-ERT2 d4 +Tam vs somatic d4 $P < 0.0001$.

© The Author(s)

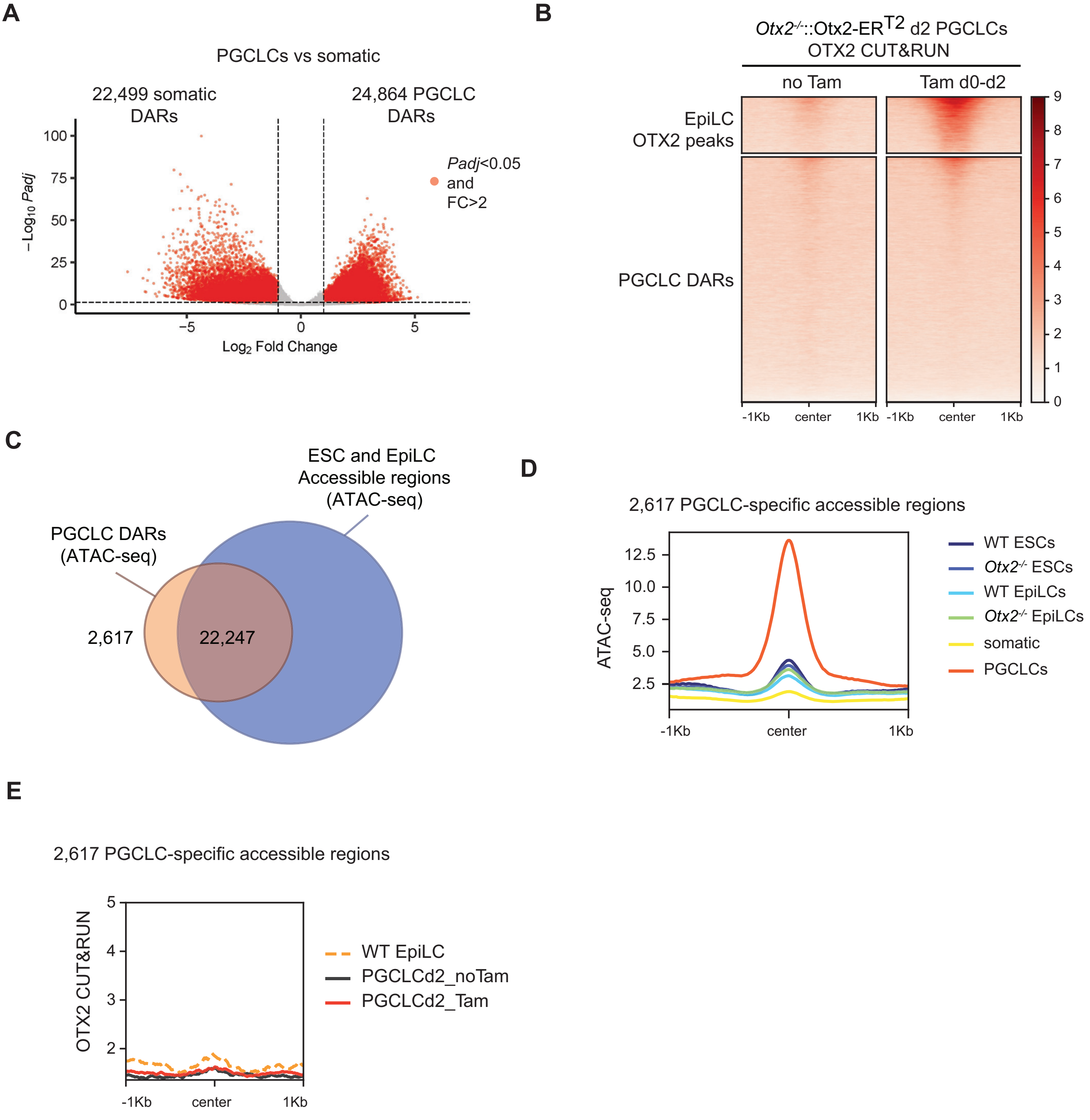

Figure EV6. OTX2 does not control accessibility of PGCLC-specific accessible regions.

(**A**) Volcano plot comparing differentially accessible regions in somatic cells and PGCLCs. Analysis performed and plot generated by DESeq2 using Wald test with Benjamini–Hochberg correction for multiple testing from $n = 2$ biological replicates per condition. (**B**) Heatmap of OTX2 CUT&RUN signal in tamoxifen-treated *Otx2*$^{-/-}$::Otx2-ER^{T2} d2 PGCLCs at EpiLC OTX2-bound regions and PGCLC DARs. (**C**) Venn diagram of the overlap of PGCLC DARs (orange) and ESC+EpiLC accessible regions identifying 2617 PGCLC-specific accessible regions. (**D**) Average read density profiles of ATAC-seq signal in wild-type and *Otx2*$^{-/-}$ ESCs, wild-type and *Otx2*$^{-/-}$ EpiLCs, PGCLCs and somatic cells at 2617 PGCLC-specific regions. (**E**) Average read density profiles of OTX2 CUT&RUN in wild-type EpiLC and tamoxifen-treated *Otx2*$^{-/-}$::Otx2-ER^{T2} d2 PGCLCs at PGCLC-specific regions.

 © The Author(s)
